# Poster 1020: Efficacy and safety of sublingual specific immunotherapy with Pru p 3 in a portuguese population - clinical and immunological evaluation during 12 months

**DOI:** 10.1186/1939-4551-7-S1-P11

**Published:** 2014-02-03

**Authors:** Ana Célia Costa, Fátima Duarte, Elisa Pedro, Alcinda Melo, Manuel Pereira Barbosa, Conceição Santos

**Affiliations:** 1 Immunoallergology Department, Hospital de Santa Maria, CHLN, Lisbon, Portugal; 2 Laboratório de Imunologia Clínica, Instituto de Medicina Molecular, Faculdade de Medicina de Lisboa, Lisbon, Portugal

## Background

Peach allergy is prevalent, persistent and potentially severe. LTPs (Pru p 3) and profilins (Pru p 4) are the main allergens involved in this kind of allergy in patients(pts) of Mediterranean area. The hidden presence of LTPs in foodstuffs and in other food involved in LTP syndrome can trigger severe reactions, including anaphylaxis. Thus, this type of allergy can be considered an important target for specific immunotherapy(IT).

## Aims

To demonstrate the efficacy and safety of sublingual IT (SLIT) with Pru p 3 in pts with systemic reactions (SR) associated with peach ingestion, by evaluation of clinical and immunological parameters during 12 months.

## Material and methods

Eight pts (7F,1M;mean age-25.6; 19-41 years) with a history of peach allergy, confirmed by Oral Food Challenge (unless anaphylaxis symptoms and evidence of peach sensitization), undergoing SLIT Pru p 3 (ALK-Abelló) during 1 year, were evaluated. 100% of pts had SR (75% anaphylaxis) associated with peach ingestion (62.5% reported symptoms with other foods containing LTPs). All patients underwent skin prick tests (SPT; Bial-Aristegui) with aeroallergens battery, peach extract peel and pulp, other foods according to symptoms, Pru p 3 and Pru p 4 (to exclude Pru p 4 sensitization). The SLIT with Pru p 3 had an induction phase (4 days in Immunoallergology Department), followed by outpatient maintenance phase. In all pts were performed SPT and quantified specific IgE and IgG4 (sIgE and sIgG4)-UniCAP (PhadiaThermofisher) for peach and Pru p 3 before(T0), 1(T1), 6(T6) and 12 months (T12) after SLIT initiation. Basophil Activation Test(BAT) was performed by Flow2 ® CAST method (Bühlmann) with Pru p 3 extract (Alk-Abelló) with three concentrations 0.05, 0.5 and 5ug/mL at the same times.

## Results

There was a significant decrease of the mean wheal diameter of SPT in T0-T12, with peel and pulp peach (p=0.0039) and Pru p 3 (p=0.0078). Quantification of sIgE to peach and Pru p 3: significant decrease of T0-T12 (p=0.0408 and p<0.001 respectively); sIgG4: significant increase of T0-T12 (p<0.0001) for the same allergens; BAT: significant decrease between the different times and in three concentrations. During the 12 months of treatment with SLIT, there were only local reactions (itching) during the induction phase in 50% of pts with spontaneous resolution.

## Conclusions

In this initial evaluation, SLIT Pru p 3 seems to be a promising and safe therapeutic option for patients with severe peach allergy.

